# Investigating ABO Blood Groups and Secretor Status in Relation to SARS-CoV-2 Infection and COVID-19 Severity

**DOI:** 10.3390/jpm14040346

**Published:** 2024-03-26

**Authors:** Stefanos Ferous, Nikolaos Siafakas, Fotini Boufidou, George P. Patrinos, Athanasios Tsakris, Cleo Anastassopoulou

**Affiliations:** 1 Department of Microbiology, Medical School, National and Kapodistrian University of Athens, 75 Mikras Asias Street, 11527 Athens, Greece; sferous@med.uoa.gr (S.F.); atsakris@gmail.com (A.T.); 2 Department of Clinical Microbiology, Attikon General Hospital, Medical School, National and Kapodistrian University of Athens, 12462 Athens, Greece; nsiaf@med.uoa.gr; 3 Neurochemistry and Biological Markers Unit, 1st Department of Neurology, Eginition Hospital, Medical School, National and Kapodistrian University of Athens, 11528 Athens, Greece; fboufidou@med.uoa.gr; 4 Laboratory of Pharmacogenomics and Individualized Therapy, Department of Pharmacy, School of Health Sciences, University of Patras, 26504 Patras, Greece; gpatrinos@upatras.gr; 5 Zayed Center for Health Sciences, United Arab Emirates University, Al Ain P.O. Box 15551, United Arab Emirates; 6 Department of Genetics and Genomics, College of Medicine and Health Sciences, United Arab Emirates University, Al Ain P.O. Box 15551, United Arab Emirates

**Keywords:** histo-blood group antigens (HBGAs), Lewis antigens, non-secretor phenotype, fucosyltransferase, *FUT2*, long COVID

## Abstract

The ABO blood groups, Lewis antigens, and secretor systems are important components of transfusion medicine. These interconnected systems have been also shown to be associated with differing susceptibility to bacterial and viral infections, likely as the result of selection over the course of evolution and the constant tug of war between humans and infectious microbes. This comprehensive narrative review aimed to explore the literature and to present the current state of knowledge on reported associations of the ABO, Lewis, and secretor blood groups with SARS-CoV-2 infection and COVID-19 severity. Our main finding was that the A blood group may be associated with increased susceptibility to SARS-CoV-2 infection, and possibly also with increased disease severity and overall mortality. The proposed pathophysiological pathways explaining this potential association include antibody-mediated mechanisms and increased thrombotic risk amongst blood group A individuals, in addition to altered inflammatory cytokine expression profiles. Preliminary evidence does not support the association between ABO blood groups and COVID-19 vaccine response, or the risk of developing long COVID. Even though the emergency state of the pandemic is over, further research is needed especially in this area since tens of millions of people worldwide suffer from lingering COVID-19 symptoms.

## 1. Introduction

Human evolution has been driven, in large part, by our constant exposure to numerous infectious agents [1]. Advantageous traits for survival that confer resistance, and, in some cases, immunity to pathogens present in our environment, are bound to be selected and passed on to future generations. Over the decades, numerous human genetic loci have been associated with resistance to infectious diseases, including genes not associated with immune function [1]. Conversely, microbial genetic analyses indicate selection for traits that can bypass human resistance. Thus, humans and infectious microorganisms co-evolve, in a constant arms race of genetic and evolutionary wits [1]. These genetic relationships are of importance because they elucidate key pathways in the pathogenesis of infectious diseases and aid us in understanding why certain infectious agents cause no symptoms in some, and severe, debilitating illnesses in others. This knowledge can help us create individualized approaches to preventing and treating infectious diseases, while also providing valuable insights into biological mechanisms in effect in health.

Studies demonstrating a biological relationship between specific ABO genotypes and the development of human diseases were published as early as the 1950s [2]. Of particular interest is the fact that the ABO and Lewis blood groups have been associated with susceptibility to numerous viral and bacterial agents. Moreover, the presence of ABO antigens in secretions and epithelial surfaces that are associated with the secretor system has also been associated with differential susceptibility to several pathogens. For example, secretors are known to be more susceptible to norovirus infections, while non-secretors are prone to severe cholera infections [3,4]. It is still unknown how different genotypes affect vaccine immunogenicity; however, studies have indicated that rotavirus vaccine efficacy, for instance, can vary with secretory status [5].

Respiratory tract infections present a challenge to healthcare systems. These infections pose a significant economic burden not only in terms of hospitalization costs, but also in terms of the costs associated with doctor visits and loss of work [6]. In addition, lower respiratory tract infections are associated with significant mortality, with pneumonia causing approximately 3 million deaths globally per year [7]. The recent ongoing severe acute respiratory syndrome coronavirus 2 (SARS-CoV-2)/coronavirus disease 2019 (COVID-19) pandemic has fueled respiratory tract infection-associated mortality. Over seven million deaths have been reported globally, as of 7 January 2024 [8].

Given the frequency of SARS-CoV-2 infections and reinfections, and the always imminent potential risk of serious COVID-19 or long COVID [9], identifying human genetic loci or genes associated with increased chances for viral transmission and disease severity is of importance. This is true not only in initial risk assessments, but also possibly in patient triage, and in the promotion of personalized preventive medicine, which will hopefully be realized in the near future. This comprehensive narrative review aims to present the current state of knowledge on the potential association of the ABO, Lewis, and secretor blood groups with SARS-CoV-2 infection and COVID-19 severity. Beyond this primary aim, we also investigate whether these blood group systems have been related to other common viral and bacterial respiratory pathogens, such as influenza viruses, *Respiratory Syncytial Virus (RSV)*, and *Mycoplasma pneumoniae*.

## 2. Methods

The PubMed database was searched for all English-language original articles or reviews using the term “blood group” alongside the viral pathogen in question, i.e., “ SARS-CoV-2”, or the resulting “COVID-19” or “long COVID”. Similar searches were conducted using the term “secretor status” instead of blood group. Paraphrased terms were also used, including “ABO-blood group”, “histo-blood group antigens”, and “secretory phenotype” or “non-secretory phenotype”. In order to explore the literature for potential associations between COVID-19 vaccine safety and efficacy and ABO blood antigens or secretor status, the term “vaccine response”, and paraphrased terms such as “vaccine immunogenicity” and “adverse events post vaccination”, were added to the initial query. The same methodological steps were applied to search the PubMed database for studies (English-language original articles or reviews) relating ABO blood antigens and secretor status with infections (or severity of infections) with influenza viruses, *Rhinovirus*, *Parainfluenza*, *Adenovirus*, *RSV*, *Mycoplasma pneumoniae*, *Chlamydia pneumoniae*, and *Legionella pneumonophila*.

## 3. Results

### 3.1. Overview of the ABO Blood Group, Lewis Antigens, and Secretor Systems

Histo-blood group antigens (HBGAs) are complex carbohydrate molecules that are expressed on the surface of red blood cells (RBCs) and mucosal epithelial cells. HBGAs are highly polymorphic, presumably providing intraspecies diversity that allows the body to cope with diverse and rapidly evolving pathogens [10]. They include the ABH, secretor, and Lewis antigens. The ABH antigens form the basis of the ABO blood group system, the main grouping system used in transfusion medicine. The basis of the ABH blood group is the H antigen, encoded indirectly by the *FUT1* gene (*H* gene) in RBCs [11] and by the *FUT2* gene (*Se* gene) in secretory cells [11,12,13,14,15]. The *H* and *Se* genes code for two homologous α-1,2-fucosyltransferases, which catalyze the addition of a fucose sugar to a galactose moiety found in oligosaccharide chains [16]. Both genes are located on chromosome 19 in close proximity to each other, as shown in Table 1, which displays the key information on fucosyltransferase 1–3 genes. Currently, there is no indication that these genes follow a clear autosomal dominant pattern of inheritance where a single copy of the mutated gene from one parent would be enough to cause the condition [11,12,13,14,15]. The addition of a fucose moiety on type 2 carbohydrate chains in RBCs by *FUT1*, and in type 1 carbohydrate chains in secretory cells by *FUT2*, results in the production of the H antigen. The H antigen is used as a precursor for subsequent A and B antigen syntheses.

The ABO gene resides in chromosome 9 and can be found in three alleles: I_A_, I_B_, and i. I_A_ and I_B_ are co-dominantly expressed and code for a glycosyltransferase responsible for A and B antigen syntheses, respectively. The i allele, which is recessive, codes for a non-functional enzyme [11]. Based on the combination of alleles an individual possesses, four blood groups can be determined: A, B, AB, and O. Individuals with an A blood group possess a functioning *H* gene and either I_A_I_A_ or I_A_i alleles. Similarly, an individual with a B blood group possesses a functional *H* gene and either I_B_I_B_ or I_B_i alleles, while an individual with an AB blood group possesses a functional *H* gene, one copy of I_A_, and one copy of I_B_. Individuals who do not possess either A or B genes but express the H antigen are classified as having type O blood. A rare blood group termed the “Bombay phenotype” refers to individuals who possess a non-functional *H* gene (designated *h* when inactive) and a non-functional *Se* gene (designated *se* when inactive), resulting in the complete absence of the H antigen both in RBCs and in exocrine secretions. Since the A and B antigens cannot be synthesized without the H antigen backbone, these individuals lack the A and B antigens as well [17]. Variations of the “Bombay phenotype” due to different polymorphic combinations are also known to exist: these include the “para-Bombay phenotype”, which is used to characterize individuals who are *h*-gene-homologous but are secretors [17].

Individuals who produce HBGAs in mucosal cells and secretions due to an active *Se* gene are called secretors [18]. Individuals with no detectable HBGAs in their secretions, as a result of having two *se* alleles, are termed non-secretors. Some individuals classified as non-secretors are actually weak secretors since small amounts of HBGAs can be found in their gastrointestinal (GI) tract and secretions. This low-level secretion stems from mutations in *FUT2* that do not encode for an inactive form of the FUT2 enzyme, but, rather, for an enzyme with reduced activity [19].

**Figure 1:**
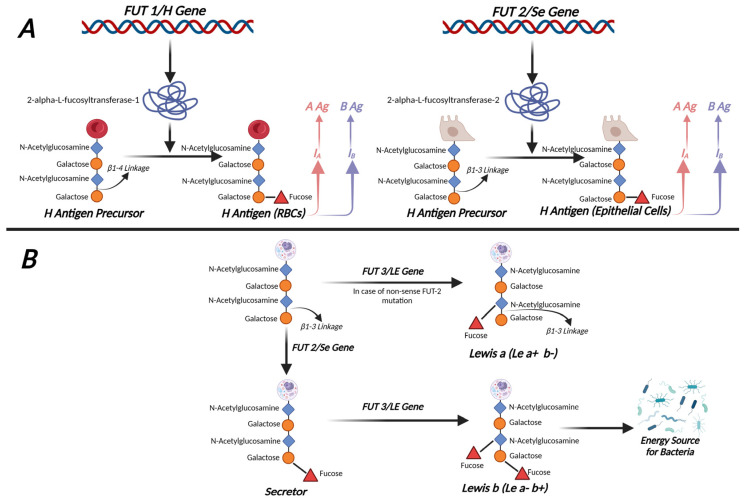
(**A**) Synthesis of ABH antigens in red blood cells (RBCs) and epithelial cells. (**B**) Synthesis of Lea and Leb from type 1 chains located in epithelial cells via *FUT3*. Adapted from [15] and created with BioRender.com. FUT1: fucosyltransferase 1, FUT2: fucosyltransferase 2, FUT3: fucosyltransferase 3, I_A_: A antigen allele, I_B_: B antigen allele, Lea: Lewis antigen A, Leb: Lewis Antigen B.

The Lewis antigens are produced by the *FUT3* gene (*Le* gene), which codes for an enzyme with both an α-1,3-fucosyltransferase and α-1,4-fucosyltransferase activity [20]. Lewis antigens (both *Lea* and *Leb*) are present in epithelial cells and in secretions because the Le enzyme acts on type 1 carbohydrate chains, which are not present in RBCs; thus, Lewis antigen synthesis does not happen in RBCs. RBCs acquire Lewis antigens via adsorption, when the cells come into contact with the soluble forms of the antigens [21]. The addition of a fucose moiety directly on type 1 carbohydrate chains by the *Le* gene results in the synthesis of the Lea antigen, whereas the addition of a fucose moiety on H antigens results in the formation of the Leb antigen [20,22]. Since the Leb antigen requires the H antigen backbone, which must be synthesized by the *Se* gene in epithelial cells and secretions, the Leb antigen is not present in non-secretors. Non-secretors, however, continue to produce Lea if they possess a functional *Le* gene [23]. Exposure to *FUT3* of the A and B antigens synthesized by *FUT2* in conjunction with I_A_ and/or the I_B_ genes results in the development of the ALeb (in A-blood individuals), or BLeb (in B-blood individuals), or both (in AB-blood individuals) [24]. An overview of the synthesis of ABH and Lewis antigens in both RBCs and epithelial cells is shown in Figure 1.

Through their roles in determining the expression of specific carbohydrate antigens in various tissues, the ABO blood group, Lewis antigens, and secretor systems are, thus, interconnected and can potentially impact disease susceptibility and immune responses to a number of rapidly evolving pathogens [10]. Herein, we review their potential associations with SARS-CoV-2.

### 3.2. Reported Associations of SARS-CoV-2/COVID-19 with ABO Blood Groups, Lewis Antigens, and Secretor Systems

SARS-CoV-2, the causal agent of the COVID-19 pandemic, is a single-stranded positive-sense RNA virus [25]. Classic symptoms of infection include fever; myalgia; rhinorrhea; cough; dyspnea; anosmia, especially in mild cases; and, in severe cases, respiratory failure due to viral pneumonia [26]. In certain cases, initial upper respiratory tract symptoms are followed by a robust inflammatory response associated with multiorgan failure, acute respiratory distress syndrome (ARDS), and systemic thrombosis [27,28,29].

Initial reports suggested that the virus was primarily transmitted through respiratory droplets produced by symptomatic individuals during coughing or sneezing. However, it is now known that asymptomatic (or minimally symptomatic) individuals can readily spread the virus through other activities, such as singing or even just by talking [30]. This appears to be especially true for children, who frequently exhibit few or no symptoms compared to adults [31]. Viral loads peak before the onset of symptoms and, thus, asymptomatic transmitters can readily spread the infection unknowingly throughout a community [27,32].

The existence of asymptomatic or pauci-symptomatic individuals has fueled research into risk factors and genetic markers associated with susceptibility to SARS-CoV-2 infection and disease severity. Obesity, arterial hypertension, smoking, diabetes, and coronary heart disease were immediately recognized as risk factors for severe disease [33,34]. Old age and being male also emerged quickly as risk factors for severe disease [35,36,37]. Similarly, numerous genetic loci have been associated with increased susceptibility to infection and increased chances of disease severity [38]. The determinants of differing susceptibility to SARS-CoV-2 infection mostly appear to entail genes related to the initial stages of infection (i.e., cell entry components such as the cell receptor angiotensin-converting enzyme 2 (ACE2) and the transmembrane serine protease (TMPRSS2) that is utilized for the priming of the spike protein of the virus). In contrast, the determinants of differing severity of COVID-19 predominantly seem to include components of the immune response to the virus (i.e., innate antiviral defense mechanisms early on in the disease and host-driven inflammatory lung injury at late disease stages) [38].

#### 3.2.1. ABO Blood Groups and Secretor Status in Relation to COVID-19 Severity

Initial reports from the onset of the pandemic indicated a link between COVID-19 severity and ABO blood groups [39], with subsequent research works reporting similar findings. In particular, blood group A patients were shown to exhibit both increased susceptibility to SARS-CoV-2 infection [38,40,41,42,43,44,45,46,47,48,49,50,51] and increased illness severity [42,47,48,52,53,54]. Increased infection susceptibility has also been reported for individuals of blood group AB [46,50], as well as for those of blood group B in addition to blood group A [47], while one study reported reduced susceptibility for individuals of the AB blood group [43]. Similar findings have been reported in children as well [55].

Maraccini et al. further demonstrated that susceptibility profiles differed between the pre- and post-Omicron eras [56]. Pre Omicron, individuals with A and AB blood were more susceptible to infection, whereas post Omicron, subjects of the O blood group were associated with an increased risk of infection. Regardless of the circulating variants of the virus, individuals of the A blood group were consistently reported to have an elevated risk of a more severe disease course [56].

It is important to note that not all studies support the association of the ABO blood subgroup with COVID-19 severity [49,57,58]. However, subsequent whole-genome sequencing and proteomics studies have associated ABO and secretor status genes with disease severity. More specifically, Kousathanas et al. used whole-genome sequencing to compare 7491 critically ill individuals with 48,400 uninfected controls; their study identified 16 new independent associations of genes that significantly predispose individuals to critical COVID-19, with variants within blood-type antigen secretor status (*FUT2*) included among them [59]. The authors concluded that predisposition to life-threatening COVID-19 can be mediated through at least two mechanisms: failure to control viral replication, or enhanced pulmonary inflammation and intravascular coagulation [59]. It is also interesting to note the association between the A blood group and the development of ARDS in non-COVID-associated conditions, such as burns [60]. Secretors, especially A-blood-type secretors, appear to suffer from higher rates of COVID-19-related morbidity and mortality [61,62].

One study of African patients with COVID-19 concluded that Le (a+b-) individuals usually exhibited mild symptoms following infection; in contrast, Le (a-b-) individuals were also associated with a lower risk of disease acquisition, but, if infected, ran a higher risk of severe disease [63]. In line with these results, Moslemi et al., using varying data on secretor status and blood groups from a large cohort of 650,156 Danish blood donors, demonstrated the reduced risk of infection in Le (a+b-) individuals [64]. Whether this is due to the presence of Lea or the absence of Leb is yet to be determined. No associations were identified between blood groups or secretor status and COVID-19 severity (indicated by the need for hospitalization) [64].

Different blood group distributions in different geographic regions or ethnicities may account for the divergent observations regarding the associations of ABO blood types, Lewis antigens, and secretor status with SARS-CoV-2 infection and COVID-19 severity [65]. Nonetheless, there seems to be consensus at least regarding the relative protective effects of blood type O in contrast to type A and possibly AB, always within the context of a seemingly infinitely diverse genetic background of a constellation of many other human polymorphisms that could also be involved, in addition to a plethora of other potentially contributing factors (e.g., comorbidities, and viral or environmental parameters) [38].

#### 3.2.2. Proposed Theories for Explaining the Potential Association between the A Blood Group and Increased COVID-19 Severity

The association between the A blood group and disease susceptibility and severity appears to hold true. Numerous theories have been proposed to explain this likely biological relationship.

First, it has been postulated that circulating anti-A antibodies, present in individuals of blood types O and B, attach to the viral S protein, thereby interfering with viral attachment to the angiotensin-converting enzyme 2 (ACE2) receptor and lowering the chances for infection [66,67,68,69,70]. Similar findings were demonstrated during the SARS-CoV-1 pandemic of 2003 [71]. Meta-analyses have identified the absence of anti-A antibodies as a risk factor for severe COVID-19 and death [72], supporting the theory that disease susceptibility and severity are not related to the presence or absence of specific blood group antigens, but rather to the presence or absence of anti-A antibodies [73]. Similar results were reported by Matzhold et al., who demonstrated that the presence of both anti-A and anti-B antibodies, as in type O blood, in Caucasian adults from Austria exerted a protective effect against COVID-19, even though they found no differences in mortality among hospitalized patients between the blood groups [65]. Apart from patients with type O blood, those with the Lewis (a-b-) blood type were also found to be significantly protected and less likely to be hospitalized due to COVID-19, in contrast to type AB subjects, who were more likely to be found in the patient cohort [65].

Second, studies have demonstrated that patients with A and AB blood are at an increased risk of having strokes, peripheral arterial disease, and myocardial infarctions [74,75]. This could possibly be due to increased leukocyte adhesion to vascular walls and increased von Willebrand factor levels, both of which promote vascular inflammation and thrombosis [76]. Therefore, SARS-CoV-2 infection could further augment an already prothrombotic state in patients of non-O blood groups [77], thus increasing thrombotic risk and overall mortality.

Third, cytokine levels over the course of COVID-19 might also differ between ABO blood groups. A study by Tamayo-Velasco et al. showed that patients of non-O blood groups maintained higher cytokine levels than their O-blood counterparts; moreover, increased levels of Hepatocyte Growth Factor were associated with increased mortality [78]. However, other studies do not corroborate these findings. Hoiland et al. measured IL-1β, IL-6, IL-10, and TNF-α levels in 125 patients with severe COVID-19, and although their study demonstrated increased risks for mechanical ventilation and overall disease severity in patients of blood groups A and AB, no significant differences in cytokine expression were found between the ABO blood groups [79].

Other theories that have been proposed to explain the likely association between the A blood group and COVID-19 susceptibility and severity include the preferential attachment of SARS-CoV-2 to the A antigen [80], as well as changes in sialic acid-containing receptors on cellular membranes induced by ABO antigens [81].

#### 3.2.3. ABO Blood Groups and Secretor Status in Relation to Long COVID

Recent epidemiological data have shown that the severity of SARS-CoV-2 infections may not be the only factor associated with the development of long COVID as was initially suspected, since patients with mild initial episodes were also found to experience lingering symptoms compatible with long COVID [82]. In addition, reinfections appear to increase the risk of the development of chronic symptoms [9,83]. Therefore, it would seem logical that A-blood individuals are at a higher risk of developing long COVID since they appear to be more susceptible to infection and usually suffer from a more severe disease course.

To date, few studies have investigated the association between ABO blood group antigens, secretor status, and long COVID. A prospective study by Soriano et al., which analyzed data from approximately 5500 patients from Spain, found no association between the ABO blood types and the development of long COVID [58].

Similarly, no associations were found between specific blood groups and the development of long COVID by Moslemi et al., who analyzed data from 36,068 blood donors who tested positive for SARS-CoV-2 from a large Danish cohort of approximately 650,000 blood donors [64]. Their study nevertheless corroborated the protective effect of blood group O [64]. The explanation behind the absence of a relationship between the A blood group and long COVID remains elusive. If A-blood individuals are indeed more susceptible to infection, then this lack of association between this specific blood group and long COVID indicates that other genetic loci or genes, possibly in combination with several other patient risk factors, such as female sex, obesity, and smoking [84], may be associated with the development of long COVID.

However, caution must be exercised when drawing conclusions about genetic susceptibility regarding an ill-defined illness such as long COVID that may present with over 200 different symptoms [82], the exact pathophysiology of which have yet to be elucidated. Personalized medicine is expected to greatly assist in understanding the underlying mechanisms behind the development of long COVID and aid in identifying high-risk individuals.

#### 3.2.4. ABO Blood Groups and Secretor Status in Relation to COVID-19 Vaccine Efficacy and Safety

Only a handful of studies have investigated the potential association between ABO blood groups and COVID-19 vaccine responses. One limited study of 85 medical students found no association between blood groups and vaccine responses [85]. Accordingly, Allan et al. showed that ABO blood groups did not affect vaccine side effects in approximately 4000 healthcare workers, students, and volunteers who received the two mRNA COVID-19 vaccines by Pfizer-BioNTech and Moderna that are available in the European Economic Area (EEA) and the United States [86]. Alessa et al. reported similar results, finding no association between blood groups and vaccination side effects [87]. Almaki et al. studied 760 adults who received at least one dose of either of the two leading mRNA vaccines or AstraZeneca’s simian adenovirus-based COVID-19 vaccine [88]. Their study reported an increased risk of severe side effects following vaccination in B-blood individuals, especially after the second dose, and predominantly in recipients of the adenovirus-based vaccine [88]. Overall, more carefully designed studies are needed to better characterize whether ABO blood groups affect COVID-19 vaccine responses in terms of the vaccines’ safety and efficacy.

## 4. Conclusions

ABO blood types, Lewis antigen expression, and the secretion of HBGAs have been implicated in the establishment and prognosis of numerous microbial infections. Investigating the relationship between blood antigens and pathogens can aid in the identification of high-risk individuals, personalize treatment, and clarify epidemiological patterns.

Our literature review identified an association between the A blood group and susceptibility to SARS-CoV-2 infection. An association between blood group A and disease severity is also frequently reported in the literature. Further exploration of a possible explanation for this mechanism has revealed that the absence of anti-A antibodies (i.e., the A blood group and possibly AB as well) is likely associated with disease susceptibility and mortality [89]. Thus, an antibody-mediated mechanism may be responsible for this association. Other mechanisms, which include differing cytokine expression profiles between the different blood groups, warrant further study.

Moreover, ABO and secretor antigens have been associated with alterations in the gut microbiota’s composition [90,91]. Gut microbiota alterations have been associated with the development of numerous diseases, ranging from autoimmune disorders to malignancies [92,93]. Thus, biochemical changes induced by different ABO antigens can explain the proclivity of certain blood groups to develop certain diseases. For example, individuals of blood group A appear to be more prone to developing cancer [94]. The normal microbiome of the lung has also been recently appreciated as a contributing factor to several respiratory diseases, including asthma and also possibly COVID-19 pneumonia [95,96]. Considering the effect that specific HBGAs have on the gut microbiome’s composition, it would be of interest to determine whether similar alterations in our lung microbiome are also dependent on blood group and secretor status.

Finally, our research found little evidence for the association of ABO blood groups, Lewis antigen expression, and secretory status with other respiratory tract pathogens, such as influenza viruses or RSV. In striking contrast with SARS-CoV-2 infection, current findings do not provide a robust connection between ABO blood types and susceptibility to infection with influenza viruses. Although a recent study concluded that individuals with A-type blood are less susceptible to influenza infection [97], this finding is not consistently reported within the literature [98]. The reports associating secretory status with influenza are also scarce, with only one, by Raza et al., demonstrating an increased risk of influenza infection amongst secretors [99]. Similarly to SARS-CoV-2, associations with responsiveness to vaccines against influenza are also rare. One study conducted in 1978 demonstrated increased rates of seroconversions following the first dose of a live-attenuated influenza vaccine in individuals with type A blood as well as a higher antibody titer following vaccination with a killed subunit vaccine in individuals with type O blood [100]. Our literature review returned no studies relating ABO blood antigens and secretor status with RSV, *Rhinovirus*, *Parainfluenza*, *Adenovirus*, *Mycoplasma pneumoniae*, *Chlamydia pneumoniae*, or *Legionella pneumonophila* infections (or the severity of infections)*.* Considering the recent approval of the novel RSV vaccines in pregnant people and older adults, further studies into these potential associations, particularly in relation to RSV, might be fruitful. Understanding the effect that specific host markers have on immune system functions and on an individual’s susceptibility to specific diseases can enhance personalized diagnostic, therapeutic, and preventative interventions in addition to elucidating the pathophysiological mechanisms behind the development of diseases.

## Figures and Tables

**Table 1:** Key information on fucosyltransferase 1–3 genes.

	*FUT1*	*FUT2* (*Secretor* Gene)	*FUT3* (*Lewis* Gene)
Other gene names	*H* gene	*B12QTL1*, *SE*, *Se2*, *SEC2*, *sej*	*Le* gene
Size (kb)	~4.00	9.98	2.37
Chromosomal location	19q13.3	19q13.33	19p13.3
Encoded enzymes	α-1,2-fucosyltransferase 1 (α2FucT1)	α-1,2-fucosyltransferase 2 (α2FucT2)	α1-4-fucosyltransferase (FucT)
Functions	Regulation of the expression of the H antigen mainly on erythrocyte membranes	Regulation of the expression of the H antigen mainly in epithelial cells and in bodily fluids such as saliva	Synthesis of the Lea and Leb antigens
Key reviews	[11,13,14]	[11,14,15]	[14]

## Data Availability

Not applicable.

## Abbreviations

ACE2	Angiotensin-converting enzyme 2
ARDS	Acute respiratory distress syndrome
COVID-19	Coronavirus disease 2019
*FUT1* (*H* gene)	Fucosyltransferase 1
*FUT2* (*Secretor* gene, *Se*)	Fucosyltransferase 2
*FUT3* (*Lewis* gene, *Le*)	Fucosyltransferase 3
HBGAs	Histo-blood group antigens
Lea	Lewis antigen A
Leb	Lewis antigen B
RBCs	Red blood cells
SARS-CoV-2	Severe acute respiratory syndrome coronavirus 2
